# What trial participants need to be told about placebo effects to give informed consent: a survey to establish existing knowledge among patients with back pain

**DOI:** 10.1136/medethics-2016-103964

**Published:** 2017-06-29

**Authors:** John Hughes, Maddy Greville-Harris, Cynthia A Graham, George Lewith, Peter White, Felicity L Bishop

**Affiliations:** 1 Royal London Hospital for Integrated Medicine, London, UK; 2 Department of Psychology, University of Southampton, Southampton, UK; 3 School of Medicine, Southampton University, Southampton, UK; 4 Department of Health Sciences, University of Southampton, Southampton, UK

**Keywords:** Informed consent, Clinical trials, Patient perspective

## Abstract

**Introduction:**

Patients require an accurate knowledge about placebos and their possible effects to ensure consent for placebo-controlled clinical trials is adequately informed. However, few previous studies have explored patients’ baseline (ie, pretrial recruitment) levels of understanding and knowledge about placebos. The present online survey aimed to assess knowledge about placebos among patients with a history of back pain.

**Design:**

A 15-item questionnaire was constructed to measure knowledge about placebos. Additional questions assessed sociodemographic characteristics, duration and severity of back pain, and previous experience of receiving placebos.

**Setting:**

Participants recruited from community settings completed the study online.

**Results:**

210 participants completed the questionnaire. 86.7% had back pain in the past 6 months, 44.3% currently had back pain. 4.3% had received a placebo intervention as part of a clinical trial and 68.1% had previously read or heard information about placebos. Overall knowledge of placebos was high, with participants on average answering 12.07 of 15 questions about placebos correctly (SD=2.35). However, few participants correctly answered questions about the nocebo effect (31.9% correct) and the impact of the colour of a placebo pill (55.2% correct).

**Conclusions:**

The findings identified key gaps in knowledge about placebos. The lack of understanding of the nocebo effect in particular has implications for the informed consent of trial participants. Research ethics committees and investigators should prioritise amending informed consent procedures to incorporate the fact that participants in the placebo arm might experience adverse side effects.

## Introduction

Placebos are an essential component of randomised controlled trials (RCTs). They are used to control for bias, contextual and psychological components of treatment and thus isolate the speciﬁc effects of the intervention under investigation. Administering placebos to patients can elicit both beneficial and adverse (‘nocebo’) effects. Many factors are now known to impact on the strength of the placebo response, including factors associated with the healthcare professional administering treatment, the patient receiving treatment and their therapeutic relationship.^1–4^ Characteristics of the intervention itself, such as medication colour, the form and frequency of administration also influence the strength of placebo response.^5–8^ Nocebo effects are typically linked to patient expectations derived from side-effect warnings and can be conditioned from previous adverse events.^9^ Common nocebo effects include nausea, stomach pains, itching, bloating, depression and sleep problems.^10^

It is important that potential trial participants know about placebo and nocebo effects. At minimum an accurate knowledge of the possible benefits and adverse effects of placebos is necessary to ensure consent to take part in an RCT is adequately informed. In addition, people’s understanding of, and attitudes towards, placebos may influence their willingness to participate in placebo-controlled RCTs^11 12^ and thus could have implications for fair access. However, information leaflets used in RCTs often provide incomplete or inaccurate information about placebos. Bishop *et al* found that only 1 of 45 participant  information leaflets used in major RCTs in the UK mentioned that placebos may elicit beneficial effects and only four mentioned that placebos can elicit adverse effects.^13^

It is necessary to assess people’s baseline knowledge of placebos (ie, before participating in any trial recruitment activities) in order to identify common gaps in knowledge and thus specify the placebo characteristics that should be prioritised for inclusion in participant information leaflets. However, little is known about the public’s knowledge of placebos and placebo effects. We surveyed people with back pain to examine current levels of placebo knowledge and identify knowledge gaps. To the authors’ knowledge this is the first such study. The objective was to inform improvements to informed consent procedures.

## Methods

### Design and measures

A web-based cross-sectional survey was conducted. Fifteen true-false items assessed knowledge of placebos (for items, see table 1). Items were developed after consulting with experts in placebo research and examining relevant literature. The questionnaire was pretested with 10 lay volunteers and modified based on their feedback. The survey also assessed demographic characteristics; experience of/sources of knowledge about placebos (to permit an initial assessment of the validity of our knowledge questionnaire); and history and severity of back pain and its impact on daily living, using the validated reliable Chronic Pain Grade Questionnaire.^14^ Participants also completed a 15-item acupuncture questionnaire (reported separately^15^).

**Table 1:** Participants’ knowledge about placebos

Item	Correct answer	Total % correct (n)	Read/heard about placebos (n=143)† % correct (n)	Not read/heard about placebos (n=64)† % correct (n)
A pill with aspirin in it is called a ‘placebo’ pill	False	98.1% (206)	98.6% (141)	96.9% (62)
The placebo effect can work because of people’s expectations	True	95.2% (200)	99.3% (142)	85.9% (55)**
Placebo treatments are only effective for people who are not very intelligent	False	96.7% (203)	97.9% (140)	93.8% (60)
Placebo treatments can help to treat pain conditions	True	79.5% (167)	82.5% (118)	71.9% (46)
Placebo treatments do not help to relieve any medical symptoms	False	72.9% (153)	76.2% (109)	65.6% (42)
Placebo pain treatments only relieve imaginary pain (ie, pain that was not real in the first place)	False	85.2% (179)	89.5% (128)	75.0% (48)**
A pill with no medicine in it is called a ‘placebo’ pill	True	91.9% (193)	97.9% (140)	78.1% (50)**
The placebo effect can work because of conditioning	True	74.3% (156)	74.8% (107)	73.4% (47)
Real changes in the brain can occur when you receive a placebo (such as the release of chemicals called opioids)	True	83.8% (176)	88.1% (126)	73.4% (47)*
Placebo effects are imaginary and have no real physical effects on our body	False	81.0% (170)	83.2% (119)	76.6% (49)
Placebo effects only occur in experiments and research trials	False	86.2% (181)	90.2% (129)	76.6% (49)**
A placebo pill can have side effects	True	31.9% (67)	34.3% (49)	28.1% (18)
The placebo effect can help us to get better during normal medical treatments	True	79.5% (167)	83.9% (120)	68.8% (44)*
The colour of a placebo pill can change how effective it is	True	55.2% (116)	60.1% (86)	43.8% (28)*
Placebo treatments are only effective for people who lie about their symptoms	False	96.7% (203)	99.3% (142)	90.6% (58)**

*p<0.05, **p<0.01, significant χ^2^.

†Three participants did not specify whether they had previously read or heard about placebos.

### Participants

We surveyed adults with a history of back pain, as back pain is prevalent^16^ and placebos have demonstrated pronounced effects in chronic pain conditions.^17^

### Procedure

Ethical approval was given by the University of Southampton Psychology Ethics Committee.

Fifteen UK universities invited staff and students to participate via email. The study was also advertised on social media sites pertaining to back pain and to local businesses. Adults who had ‘had back pain’ were invited to take part in a ‘short (10 min) online quiz’ about alternative treatments for back pain. On clicking a link participants reached an information page presenting study details and a tick-box to indicate consent. Participants then completed the survey.

Data were imported into SPSS V.22 and summarised using descriptive statistics. Analysis of variance, t-test, χ^2^ test and Spearman’s correlation test assessed whether knowledge of placebos was related to previous experience of receiving placebos, having read or heard about placebos, gender, age, ethnicity, highest qualification and back pain characteristics.

## Results

### Participant characteristics

Two hundred and twenty-six people participated between July and October 2014. Data were excluded from 16 individuals who failed to complete any placebo knowledge items, leaving 210 participants. One hundred and thirty-six participants were female (67.7%) and 65 were male (32.3%), aged 18–74 years (M=35, SD=14.05) (9 skipped these items). All participants reported back pain: 100% ever had back pain, 86.7% (n=182) in the past 6 months and 44.3% (n=93) currently. Of those reporting current back pain, average pain intensity was mild (M=3.4, SD=2.16) (see table 2 for additional characteristics).

**Table 2:** Participant characteristics

	Number (n)	Per cent (%)
Gender*		
Female	136	67.7
Male	65	32.3
Ethnic origin		
White British	137	65.2
Other White background	39	18.6
Asian or Asian British	9	4.3
Chinese	7	3.3
Other	16	7.7
Preferred not to state ethnicity	2	1.0
Occupation		
Student	66	31.4
Administrator/secretary	27	12.9
Academic	25	11.9
Postgraduate student	21	10.0
Researcher	19	9.0
Teaching	12	5.7
Healthcare professional	8	3.8
Currently not working/retired	7	3.3
Technician/programmer	7	3.3
Care work	4	1.9
Engineering	2	1.0
Other	12	5.7
Highest level of education		
Secondary school	10	4.8
Some college	31	14.8
Bachelor’s degree	50	23.8
Master’s degree	58	27.6
Doctoral degree	44	21.0
Other	17	8.1

Pain in past 6 months†	Mean	SD
Intensity	4.23	1.96
Interference in daily activities	3.83	2.57
Interference in recreational activities	3.08	2.70
Interference in work activities	2.93	2.61

*Nine participants did not specify their gender.

†Items answered on a 0–10 scale, where 10 indicates highest levels of pain intensity/interference.

### Experiences of placebos

Only nine participants (4.3%) had previously received a placebo as part of an RCT, but 68.1% (n=143) reported having previously read or heard about placebos, via friends and family, school/university, general knowledge, books, media and/or the internet.

### Knowledge

Participants answered between 4 and 15 knowledge items correctly (M=12.07; SD=2.35) (see table 1). Key gaps in placebo knowledge were identified; 31.9% knew that a placebo pill can have side effects and 55.2% knew that the colour of a placebo pill can change how effective it is.

The nine participants who had previously received placebo treatment as part of an RCT (M=12.22, SD=1.64) had similar knowledge scores to the 201 who had not (M=12.06, SD=2.39) (p=0.841). However, the 143 participants who reported previously reading or hearing about placebos had significantly higher scores (M=12.55, SD=2.15) than the 67 who indicated they had not read or heard about placebos (M=10.97, SD=2.49) (t=4.663, df=205, p<0.001).

There were just two differences in knowledge by sociodemographic and clinical characteristics. Participants who identified as white British had higher placebo knowledge scores than other ethnicities combined (M=12.47 (SD=2.18) and M=11.33 (SD=2.50), respectively; t=3.422, df=208, p=0.001). Participants who reported less intense pain during the previous 6 months had higher placebo knowledge scores than those who reported more intense pain (r_s_=−0.210; p<0.01).

## Discussion

Placebos are an important component of RCTs used to elucidate the speciﬁc effects of an intervention under investigation. For informed consent to be valid, trial participants need an accurate knowledge of placebos; this should minimally include an understanding that placebos can have both beneficial and adverse effects.^18^ Our community-based survey of people with back pain found relatively high knowledge overall but only a small minority of participants knew that placebos could have adverse, that is, nocebo, effects. Evidence from meta-analysis suggests as many as 52% of RCT participants receiving a placebo may experience nocebo effects.^19^ However, just 31.9% of our participants knew that a placebo can have side effects. Earlier studies elsewhere reported similar findings: 4.8% of general practitioner patients in New Zealand agreed that placebos can cause bad side effects^20^ and 7.7% of patients recruited from a rheumatology clinic  in France believed placebos can induce adverse effects.^21^ Our study updates and extends this work, suggesting that the UK patients would also benefit from receiving information about nocebo effects before taking part in a placebo-controlled RCT.

A lack of placebo knowledge among potential trial participants has implications for the ethical principle of autonomy, and consequently participants’ ability to provide full informed consent. Respect for autonomy requires potential participants to have sufficient information to enable them to make an informed decision regarding participation. In particular, the Declaration of Helsinki^18^ requires volunteers to be informed about the potential benefits and harms of participation. The knowledge gaps identified within the present study, combined with the limited descriptions of placebos in participant information sheets found previously,^13^ suggest that in many cases participants do not have an adequate understanding of the potential benefits and harms of placebos before consenting to placebo-controlled RCTs. This would appear to violate the principle of autonomy, and may question the ethical validity of consent.^22^

There is increasing awareness that ethical practices, such as the content of participant information sheets, should be grounded in empirical data.^23 24^ However, there remains a dearth of published research to inform investigators and research ethics committees. This study was strengthened by using evidence-based items to assess placebo knowledge objectively. The fact that participants who had previously read or heard about placebos scored higher than other participants provides initial evidence for the construct validity of the knowledge questionnaire.

Selection bias is a limitation; participants were more highly educated than the general UK population (almost 50% possessed a postgraduate qualification). This may have driven the high placebo knowledge scores and a more representative sample might have exhibited less knowledge; indeed, even lower levels of knowledge about nocebo effects have been reported by others.^20 21^ However, educational attainment was not related to placebo knowledge in this sample.

Research ethics committees and investigators should prioritise amending informed consent documentation and procedures to explain that participants in the placebo arm might experience beneficial and adverse effects. Our findings suggest that while volunteers may have some existing knowledge that placebos can elicit beneficial effects, they are far less likely to appreciate their potential to elicit adverse effects. Adding information about nocebo effects to participant information sheets and associated discussions might therefore increase participants’ capacity to provide ethically valid informed consent. Future research could evaluate placebo knowledge gaps in other patient groups and develop resources and guidelines to improve the provision of patient information about placebo and nocebo effects. In the meantime, we recommend that research ethics committees apply greater scrutiny to the description of placebos in participant information sheets.
